# RNA m5C modification: from physiology to pathology and its biological significance

**DOI:** 10.3389/fimmu.2025.1599305

**Published:** 2025-04-30

**Authors:** Xi Chen, Yixiao Yuan, Fan Zhou, Xiaobing Huang, Lihua Li, Jun Pu, Yong Zeng, Xiulin Jiang

**Affiliations:** ^1^ Key Laboratory of Neurological and Psychiatric Disease Research of Yunnan Province, The Second Affiliated Hospital of Kunming Medical University, Kunming, China; ^2^ NHC Key Laboratory of Drug Addiction Medicine, Kunming Medical University, Kunming, Yunnan, China; ^3^ Department of Medicine, UF Health Cancer Center, University of Florida, Gainesville, FL, United States; ^4^ Department of Hematology, the Second Hospital Affiliated to Kunming Medical University, Kunming, Yunnan, China

**Keywords:** 5-methylcytosine, methods, physiology, pathology, biological significance, cancer immunotherapy

## Abstract

RNA 5-methylcytosine (m5C) modification is a crucial epitranscriptomic mark that regulates RNA stability, processing, and translation. Emerging evidence highlights its essential role in various physiological processes, including cellular differentiation, stem cell maintenance, and immune responses. Dysregulation of m5C modification has been implicated in multiple pathological conditions, particularly in cancer, neurodegenerative disorders, and metabolic diseases. This review provides a comprehensive overview of the molecular mechanisms governing m5C deposition, its functional consequences in normal physiology, and its contributions to disease pathogenesis. Furthermore, we discuss the potential of m5C as a biomarker and therapeutic target, offering new insights into its biological significance and clinical relevance.

## 1 Introduction

To date, over 170 types of methylation modifications have been identified in RNA, including N6-methyladenosine (m6A) (1), 5-methylcytosine (m5C) (2), and 7-methylguanylate (m7G) (3). These modifications increase RNA complexity by affecting RNA tertiary structure, biogenesis, localization, and function, which are critical for cellular biological processes and cancer development. m5C methylation refers to the addition of a methyl group to the 5th carbon of the cytosine ring in DNA or RNA, which is a highly concentrated and reversible epigenetic modification (4). This modification was first discovered in DNA and later in RNA. RNA m5C modifications have widespread target sites, including messenger RNA (mRNA) and non-coding RNA (ncRNA), such as transfer RNA (tRNA), ribosomal RNA (rRNA), micro RNA (miRNA), small nuclear RNA (snRNA), and enhancer RNA (eRNA) (5).

With the continuous improvement of methylated RNA immunoprecipitation sequencing and liquid chromatography-mass spectrometry techniques, m5C modifications in mRNA have been found to affect various biological processes, such as mRNA stability, translation, splicing, and nucleocytoplasmic transport; DNA damage repair; cell proliferation and migration; and stem cell development, differentiation, and reprogramming (6–8). Previous research primarily focused on DNA, while studies on the function and regulatory mechanisms of m5C modifications in RNA are still in the early stages. In recent years, the development of methylation sequencing technologies has confirmed the presence of m5C methylation modifications in both coding and non-coding RNAs. RNA m5C methylation modifications rely mainly on methyltransferases (writers), demethylases (erasers), and binding proteins (readers) (9). Aberrant mRNA m5C modifications are associated with cancer, autoimmune diseases, and atherosclerosis (10).

In summary, 5-methylcytosine modification plays a crucial role in regulating gene expression, maintaining genomic stability, and influencing cellular differentiation. Its dynamic regulation, mediated by DNA methyltransferases and demethylases, ensures proper cellular function under physiological conditions. However, aberrant 5mC patterns are frequently associated with various pathological states, including cancer, neurological disorders, and autoimmune diseases. Understanding the mechanisms governing 5mC modification and its biological significance not only provides fundamental insights into epigenetic regulation but also offers potential therapeutic strategies for disease intervention. Future research should focus on deciphering the context-specific roles of 5mC and developing targeted approaches to modulate its function in disease treatment.

## 2 Regulatory mechanisms of RNA m5C methylation

RNA m5C methylation is a dynamic and reversible process, primarily regulated by three factors: m5C methyltransferases, demethylases, and m5C methylation binding proteins. RNA m5C methyltransferases mainly include NOL1/NOP2/sun (NSUN) methyltransferases and DNA methyltransferase-like DNMT2, which catalyze the formation of 5-methylcytosine (11). m5C methylation binding proteins function by recognizing and binding to m5C methylation sites, while demethylases catalyze the demethylation of RNA m5C.

### 2.1 m5C methyltransferases

m5C methyltransferases use S-adenosyl-L-methionine (SAM) as the methyl donor to transfer the methyl group to cytosine, forming 5-methylcytosine. Over ten RNA m5C methyltransferases have been identified, including the NSUN family, DNMT2, and the tRNA-specific methyltransferase TRDMT family (12, 13). The NSUN family proteins contain a Rossmann fold catalytic domain and a SAM binding site. Members of the NSUN family include NSUN1-NSUN7 (14). NSUN1 directly binds to the 60-80S ribosomal precursor and catalyzes the m5C modification of human 28S rRNA. NSUN2 is the most extensively studied NSUN family member (15). It can catalyze the m5C methylation modification of various RNAs, including rRNA, tRNA, mRNA, mitochondrial RNA, and viral RNA. NSUN2-mediated m5C mRNA is widely distributed across all coding regions. NSUN2 performs various biological functions, such as regulating epithelial cell differentiation, HIV-1 transcription, and EB virus degradation (16). NSUN2 is highly expressed in several tumors, mediating tumorigenesis and progression. For instance, in gallbladder cancer, silencing NSUN2 inhibits the proliferation and tumor formation of gallbladder cancer cells (17). In liver cancer, the long non-coding RNA (lncRNA) H19 is a specific target of NSUN2. m5C-modified H19 promotes liver cancer development by recruiting Ras-GTPase-activating protein SH3 domain-binding protein 1 (G3BP1) (18). NSUN3 is mainly localized to the mitochondria, catalyzing the methylation of the anticodon loop C34 site of mitochondrially encoded tRNA methionine (mt-tRNAMet) (19). NSUN4 is an rRNA-specific methyltransferase transported to the mitochondria in an N-terminal 26 amino acid motif-dependent manner (20). NSUN4 interacts with mitochondrial regulatory factor MTERF4, recruiting the mitochondrial large ribosomal subunit to promote mitochondrial ribosome assembly by methylating the 12S rRNA C911 site. NSUN5 is localized to the nucleolus and is also an rRNA-specific methyltransferase, catalyzing the methylation of the C2278 site in the IV domain of 25S rRNA (21). In colorectal cancer, highly expressed NSUN5 promotes tumor cell proliferation by regulating the cell cycle (22). NSUN6 is partially localized to the Golgi apparatus and centrosome and is a tRNA methylation regulator, catalyzing the methylation of C72 site tRNACys and tRNAThr, affecting tRNA biogenesis (23). NSUN6 expression is downregulated in tumors, and high NSUN6 expression is associated with better prognosis in some cancers (24). NSUN7 mediates the m5C methylation modification of enhancer RNA (eRNA) (25). DNMT2 possesses the sequence and structural characteristics of DNA methyltransferases and can catalyze cytosine DNA methylation (26). Additionally, DNMT2 catalyzes the methylation of C38 site tRNAAsp. DNMT2-catalyzed tRNA methylation plays important roles in tRNA processing, maintaining translation accuracy, stability, and differentiation, and protects against ribonuclease cleavage (27). Two other methyltransferases, TRM4A and TRM4B, specifically catalyze tRNA m5C methylation. In summary, methyltransferases are key regulatory factors of RNA m5C methylation, catalyzing the methylation of various RNAs (28). Although some studies have confirmed the crucial roles of methyltransferases in certain tumors, their roles and mechanisms in different tumor types remain to be elucidated.

### 2.2 m5C demethylases

The ten-eleven translocation (TET) family of demethylases is Fe(II) and α-ketoglutaric acid (αKG)-dependent dioxygenases, including TET1, TET2, and TET3 (29, 30). TET3 is distributed in both the nucleus and cytoplasm, while TET1 and TET2 are mainly localized to the nucleus (31). The TET enzyme family can catalyze the oxidation of DNA 5-methyl-2’-deoxycytidine (5mdC) to form 5-hydroxymethyl-2’-deoxycytidine (5hmdC), 5-formyl-2’-deoxycytidine (5fdC), and 5-carboxyl-2’-deoxycytidine (5cadC) (32). TET enzymes also act as RNA demethylases, exhibiting activity on 5-methylcytidine (5mrC) and its oxidative derivatives in coding and non-coding RNAs, including 5-hydroxymethylcytidine (5hmrC), 5-formylcytidine (5frC), and 5-carboxycytidine (5carC) (33, 34). The TET family can catalyze various nucleic acid substrates, including dsDNA, ssDNA, ssRNA, and DNA-RNA hybrids (35). However, further research is needed to understand the structure and biological functions of TET enzymes and how to enhance the specificity and selectivity of TET-mediated oxidation.

### 2.3 m5C methylation binding proteins

The biological functions of RNA modifications are primarily associated with their binding proteins. The main m5C methylation binding proteins are ALYREF (Aly/REF export factor) and YBX1 (Y-box binding protein 1). ALYREF is a key component of the mRNA transport protein complex TREX (36). During mRNA nuclear export, ALYREF is first recruited to bind to the 5’ end of mRNA mediated by CBP80 and to the 3’ end mediated by PABPN1 (37). ALYREF further strengthens its binding to mRNA through direct interaction with the 3’ end processing factor CstF64. In human HeLa cells and mouse tissues, ALYREF directly binds to mRNA m5C sites, promoting mRNA nucleocytoplasmic shuttling, with the binding affinity and nuclear export process mediated by NSUN2 (38). YBX1 is a newly discovered m5C binding protein that regulates mRNA stability in the cytoplasm. In bladder cancer, YBX1 recognizes and binds to m5C-modified mRNA through the indole ring of W65 in its cold-shock domain (CDS), stabilizing m5C-modified mRNA, thereby regulating mRNA metabolism (39–41). In lung cancer, YBX1 promotes tumor cell invasion, migration, and angiogenesis by directly binding to lncRNA LINC00312 (42). Recently, a notable study identified another novel RNA m5C methylation binding protein, SRSF2, and revealed its association with leukemia development. Further research found that the SRSF2P95H mutation in leukemia inhibits SRSF2 recognition of m5C, affecting mRNA alternative splicing mediated by SRSF2, and leukemia patients with impaired SRSF2-m5C binding have poor prognosis (43). YBX2 has recently been reported as a novel mammalian m5C-binding protein capable of undergoing liquid-liquid phase separation (LLPS) both *in vivo* and *in vitro* (44, 45). Other methylation binding proteins remain to be discovered and validated, and their regulatory mechanisms on RNA m5C modifications require further investigation.

## 3 The impact of m5C methylation on RNA

### 3.1 Impact of m5C methylation on mRNA

**Figure 1:**
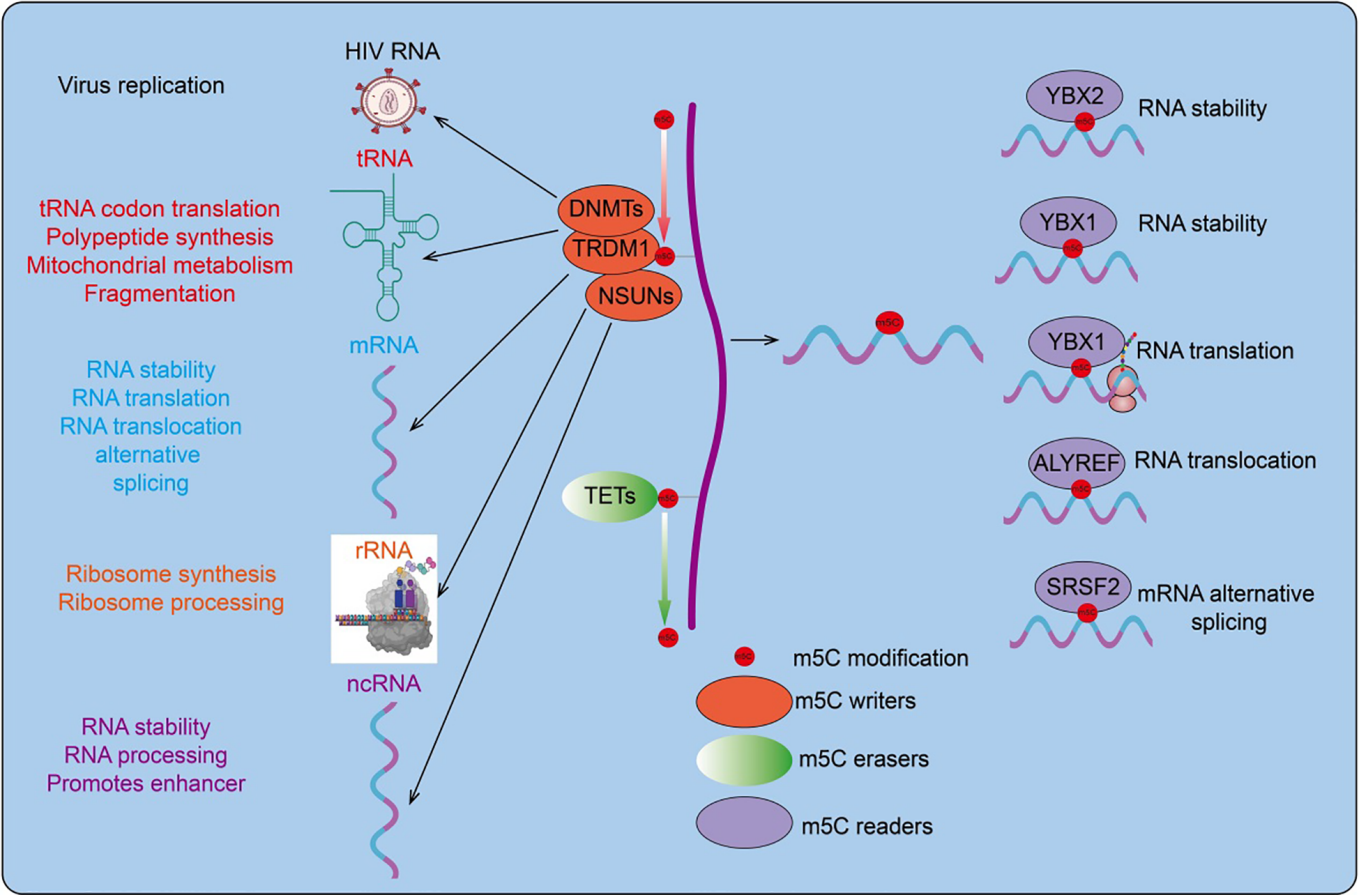
RNA m5C modification is a dynamic process. RNA m5C modification (5-methylcytosine modification) refers to the chemical modification where the cytosine residues in RNA molecules are methylated at the carbon 5 position. This modification is widely present in various types of RNA, including messenger RNA (mRNA), ribosomal RNA (rRNA), transfer RNA (tRNA), and non-coding RNA (ncRNA). RNA m5C modification plays a crucial role in various biological processes.

Extensive m5C methylation is present in mRNA, and its influence on mRNA function has become a research focus in recent years (46, 47). (1) Impact on mRNA translation: recent studies have shown a functional interdependence between m5C modifications and mRNA translation. However, m5C appears to have different effects depending on its location - specifically, it generally has negative effects in coding regions but can have positive effects in untranslated regions like the 3’-UTR. For example, in HeLa cells, m5C sites within coding regions are negatively correlated with translation efficiency (48). Another study demonstrated that NSUN2-induced m5C methylation, in collaboration with METTL3/METTL14-induced m6A methylation, mediates the methylation of the 3’ -UTR of p21 mRNA, enhancing its translation efficiency (49). (2)Impact on mRNA Transport: Research has shown that m5C modifications are enriched in CG-rich regions and downstream of the start codon, playing a critical role in mRNA nuclear export (50). (3)Impact on mRNA Stability: In bladder cancer, YBX1 enhances the stability of m5C-modified mRNA by recruiting ELAVL1 (2). However, other studies have found no correlation or a negative correlation between m5C modification levels and mRNA stability (**Figure 1**). Thus, the effect of m5C methylation on mRNA stability remains to be further investigated.

### 3.2 Impact of m5C methylation on tRNA

m5C methylation regulates tRNA stability, cellular metabolism, and stress response. Studies have shown that m5C modifications mediated by NSUN2 and DNMT2 maintain tRNA stability and regulate cellular metabolism (14). In humans and mice, TRM4/NSUN2-mediated m5C methylation prevents tRNA degradation due to oxidative stress. DNMT2-mediated tRNA methylation protects tRNA from nucleases and regulates the stability of tRNAAsp-GTC and tRNAGly-GCC (51, 52) (**Figure 1**).

### 3.3 Impact of m5C methylation on rRNA

m5C methylation regulates rRNA stability and ribosome synthesis. In the small subunit 12S rRNA, the m5C methyltransferase NSUN4 methylates cytosine 911 (m5C911) and forms a complex with MTERF4, ensuring the assembly of mature large and small subunit complexes (53). Loss of m5C2278 and G2288 methylation results in structural changes in 25S rRNA. When cells are exposed to hydrogen peroxide, the absence of Rcm1/NSUN5 leads to a more relaxed folding of sequences near 25S rRNA C2278, indicating that Rcm1/NSUN5 is crucial for maintaining rRNA stability under oxidative stress conditions (54) (**Figure 1**).

### 3.4 Impact of m5C methylation on other RNAs

m5C methylation also plays significant roles in viral RNA and lncRNA. For example, Recent studies have found that RNA cytosine-C(5)-methyltransferase (NSUN2) is upregulated in gastric cancer. NSUN2 enhances the expression of the long non-coding RNA NR_033928 through methylation modification. NR_033928, in turn, interacts with the IGF2BP3/HUR complex to upregulate the expression of glutaminase (GLS), thereby increasing the stability of GLS mRNA and promoting the progression of gastric cancer (55). m5C methylation in the interaction regions of lncRNA HOTAIR and XIST with chromatin-modifying complexes can affect XIST function by influencing its binding to the PRC2 complex (56). Viral RNAs exhibit extensive m5C methylation. Studies have shown that nucleolar protein NOP2/NSUN1 has been identified as an HIV-1 restriction factor. Functional studies confirm that NOP2 restricts HIV-1 replication. Depletion of NOP2 promotes the reactivation of latent HIV-1 proviruses in various cell lines and primary CD4+ T cells. Mechanistic studies show that NOP2 binds to the HIV-1 5’ LTR and competes with HIV-1 Tat protein for interaction with HIV-1 TAR RNA, facilitating the m5C methylation of TAR (57) (**Figure 1**). In summary, m5C methylation is widespread across various RNA types and may play crucial roles in their function. Current research on the impact of m5C methylation on RNA is still limited and contentious, necessitating further investigation.

## 4 Methods for detecting RNA m5C methylation

Current methods for detecting RNA m5C modification primarily include the following: (1) physicochemical methods, such as chromatography, mass spectrometry (MS), high-performance liquid chromatography (HPLC), and liquid chromatography-tandem mass spectrometry (LC-MS/MS); (2) chemical conversion methods combined with next-generation sequencing (NGS) technologies, such as RNA bisulfite sequencing (RNA-BisSeq) and Tet-assisted oxidation sequencing using tungsten acid (Tawo-seq); (3) immunoprecipitation combined with NGS technologies, such as 5-aza-seq (miCLIP) with m5C-specific single-nucleotide crosslinking and immunoprecipitation; and (4) third-generation sequencing (TGS) based on differential electric signal, such as Nanopore-seq (58). In practice, the most commonly used methods in research are the three described below, which we will primarily focus on, discussing their advantages, disadvantages, and other relevant aspects.

### 4.1 m5C MeRIP-seq

This method allows the examination of gene m5C methylation levels across the entire transcriptome, as well as at the tRNA level. The technical principle is as follows: m5C-specific antibodies are incubated with randomly fragmented RNA, capturing the methylated fragments for sequencing. A parallel sequencing of a control (Input) sample is also performed. The control sample consists of RNA fragments that have not undergone immunoprecipitation (IP). This control helps eliminate background noise from non-specific binding of methylated fragments. By comparing the sequencing fragments from the immunoprecipitation (IP) sample and the Input sample, m5C RNA methylation sites can be mapped to the transcriptome, allowing the calculation of m5C methylation levels in the sample.

### 4.2 m5C BS-seq

Earlier RNA m5C modification detection primarily relied on bisulfite sequencing (BS-seq). In BS-seq, unmodified cytosine (C) is converted into uracil (U), whereas m5C remains unchanged. Therefore, m5C modifications can be identified by detecting the unconverted C. Although BS-seq is straightforward and convenient, and can achieve single-base resolution of m5C modification quantification, there are three main drawbacks: 1) It detects m5C indirectly, relying on efficient conversion of unmodified C. Incomplete conversion can lead to false positives; 2) Harsh reaction conditions may cause RNA degradation, limiting detection in samples with low starting amounts or low-abundance RNA; 3) The conversion of C to U reduces sequence complexity, which affects alignment accuracy and limits m5C detection in low-complexity RNA sequences (59).

### 4.3 m5C-TAC-seq (m5C detection strategy enabled by TET-assisted chemical labeling)

The core principle of this technique is to combine enzymatic reactions with chemical labeling. The optimized TET enzyme reaction oxidizes RNA m5C to f5C, which is then specifically labeled using azidophenylfluorone (AI). This labeling product not only results in a C-to-T transition but also allows for enrichment through click chemistry, enabling direct detection of m5C modification at single-base resolution. m5C-TAC-seq can be applied to various types of RNA, including low-abundance, low-sequence complexity, and low-modification m5C sites. Additionally, it allows for the dynamic detection of m5C modifications in multiple biological processes, thus contributing to the understanding and exploration of the biological functions of RNA m5C modifications (60).

These methods each have distinct advantages and limitations, with m5C MeRIP-seq being widely used for its comprehensiveness, BS-seq providing straightforward quantification, and m5C-TAC-seq enabling high sensitivity for low-abundance RNA modifications.

## 5 The role of m5C in normal physiological processes

5-methylcytosine (m5C) plays a crucial role in normal physiological processes by regulating various aspects of RNA metabolism and gene expression. Here, we summarize the current understanding of the functions of m5C modification in neurodevelopment, autoimmune diseases, spermatogenesis, and embryonic development.

### 5.1 The role of m5C in neurodevelopment

Mutations in the m5C methyltransferase NSUN2 result in microcephaly and other neurological abnormalities in mice and humans, such as behavioral defects, speech delay, gait abnormalities, growth retardation, unusual appearance, and skin anomalies (61). In mice, the absence of NSUN2 leads to impaired neurodevelopment, inhibition of neuronal migration, and disrupted neural stem cell differentiation, causing the accumulation of intermediate progenitors and the loss of upper-layer neurons in the developing cortex (62). In Drosophila, loss of the NSUN2 homolog results in severe short-term memory deficits. Studies have found that angiogenin binds with higher affinity to tRNA lacking site-specific NSUN2-mediated methylation. The loss of m5C methylation increases angiogenin-mediated tRNA nuclear cleavage, leading to the accumulation of 5′tRNA-derived fragments, reducing protein translation rates, activating stress pathways, and causing reduced cell volume and increased apoptosis in cortical, hippocampal, and striatal neurons (63). In addition, inhibition of angiopoietin during embryogenesis can rescue the increased sensitivity of NSun2-deficient brains to oxidative stress (63). Studies have shown that loss of NSun2 function caused by autosomal recessive mutations is associated with human neurological abnormalities. Specifically, reduced NSun2 protein expression and an increased pTau/NSun2 ratio have been observed in the brains of Alzheimer’s disease (AD) patients (64). Conditional knockout of NSun2 in mouse brains promotes a decrease in m6A levels of miR-125b and excessive phosphorylation of tau. Moreover, neuronal NSun2 levels are reduced by amyloid-β oligomers (AβO). Interestingly, AβO-induced tau phosphorylation and cytotoxicity in human neurons can be rescued by NSun2 overexpression (64).

### 5.2 The roles of m5C methylation in spermatogenesis

The m5C modifications mediated by the NSUN family play a significant role in various aspects of testicular differentiation and embryonic development (65, 66). For example, the absence of NSUN2 can lead to multiple mitotic disorders and multipolar spindles, resulting in cell death (67). Studies have shown that NSUN2 deficiency leads to reduced testis size, decreased spermatogonia count, and lack of mature sperm in mice (68). Further research has revealed that NSUN2 deficiency blocks the first meiotic division and induces apoptosis in pachytene spermatocytes (69). Another member of the NSUN family, NSUN7, is also highly expressed in the testes. Its absence results in decreased sperm motility and abnormal movement, ultimately causing infertility in mice (70). Additionally, mutations in NSUN7 have been found in patients with asthenozoospermia, leading to infertility (71). The m5C modification can be inherited by offspring and is crucial for mediating acquired traits (72). Research indicates that elevated levels of m5C and m2G modifications in tRNA-derived small RNAs (tsRNAs) in sperm from high-fat diet-fed male mice affect the formation of these tsRNAs, enabling the offspring to inherit the paternal high-fat phenotype. However, the mechanism by which m5C modifications regulate tsRNA formation in sperm from high-fat diet-fed male mice remains unclear (72). Studies have demonstrated that m5C modifications mediated by DNMT2 are involved in regulating the acquired high-fat phenotype in the offspring of high-fat diet-fed mice (73). These findings suggest that abnormal m5C modifications can be inherited by offspring, leading to phenotypic changes. However, the exact mechanism of this inheritance is not yet understood, necessitating further experiments to elucidate the role of m5C in spermatogenesis regulation and epigenetics.

### 5.3 The roles of m5C methylation in embryonic development

The m5C methylation modifications mediated by NSUN family proteins have been extensively studied in the regulation of embryonic formation. Initially, researchers demonstrated the presence of NSUN2 to NSUN7 in early mouse embryos and analyzed their roles and expression patterns in embryonic development (65). They found that the m5C levels in six different animals (mice, humans, zebrafish, fruit flies, Xenopus tropicalis, and Xenopus laevis) were high during the early embryonic stages but sharply declined after the maternal-to-zygotic transition (MZT), remaining at low levels during subsequent developmental stages (74). The absence of m5C methylation modifications in early embryos leads to delayed cell cycles, preventing the timely initiation of the MZT process (74). DNMT2, another methyltransferase for m5C modifications, when singly deficient, results in neonatal mice phenotypes similar to those with dual deficiencies in NSUN2 and DNMT2, exhibiting immature hematopoietic systems, reduced numbers of hematopoietic stem cells and progenitor cells, and defects in cell-autonomous differentiation (75). However, its regulatory role in other animal embryonic developments has not yet been reported. The loss of the m5C recognition protein YBX1 disrupts zebrafish embryo cleavage and MZT processes, resulting in zygotic death post-fertilization (76). Further studies revealed that YBX1 influences normal embryonic development by inhibiting maternal mRNA translation (77). During the MZT process in zebrafish, YBX1 preferentially recognizes m5C-modified mRNA, maintaining its stability and inhibiting the translation of the maternal mRNA pool (76). The loss of YBX1 affects transcriptional activity during zygotic genome activation (ZGA) in goats and mice, with abnormal expression of splicing factors and mRNA decay genes in embryos, indicating that YBX1 impacts maternal mRNA decay, selective splicing, and transcriptional activity necessary for early embryonic development (78). This, in turn, affects early embryonic development. In Drosophila melanogaster, the Drosophila Tet homolog gene cg43444 (dtet) is positively correlated with hm5C levels. Dtet-deficient flies survive the larval stage but die during pupation, suggesting that dtet-mediated hm5C plays a regulatory role in embryonic development (79). This also indirectly highlights the importance of m5C methylation modifications in embryonic development.

## 6 The role of m5C in cancer

Studies have shown that RNA m5C modification plays an important role in cancer progression and remodeling of the tumor immune microenvironment by influencing RNA stability and translation efficiency. Therefore, in this manuscript, we systematically summarized the expression and function of RNA m5C modification in tumors, which will help us understand the occurrence and development of tumors and provide new potential targets for cancer therapy (**Tables 1**, **2**).

**Table 1:** The functional roles and mechanisms of m5C modification regulators in different cancer types: a systematic summary.

Cancer type	m5C regulators	expression	Target genes	Molecular functions	Potential mechanisms	Ref
Glioma	NSUN2	up	ATX	Enhances glioma cells proliferation	Enhancing ATX mRNA translation	(82)
LC	NSUN6	up	NH23-H1	Inhibits cell proliferation, migration and EMT in LC	Controls NM23-H1 expression by modifying the 3′-UTR of NM23-H1 mRNA using m5C.	(89)
LC	NSUN2	up	NRF2	Governs NRF2-induced ferroptosis resistance in NSCLC	Maintains the expression of NRF2 via YBX1 in NSCLC cells	(87)
LC	NSUN2	up	QSOX1	Causes gefitinib resistance and cancer recurrence in NSCLC	Regulates YBX1 and QSOX1 in NSCLC	(2)
LC	ALYREF	up	YAP1	Enhances tumor progression in NSCLC	Interacts with LINC02159; increase the stability of YAP1 mRNA; activates Hippo and beta-catenin	(37)
ESCC	NSUN2	up	GBR2	Enhances oncogenesis and progression in ESCC	Enhances m5C modification of GRB2 mRNA and its stability; activates ERK/MAPK, PI3K/AKT	(90)
GC	NSUN2	up	LINC00324	Facilitates tumor angiogenesis in GEC	Induces LINC00324 stability through m5C modification; decreases CBX3 mRNA degradation; increases VEGFR2 transcription	(134)
GC	NSUN2	up	ERK1/2	Promotes chemosensitivity in GC	Increases ERK1/2 phosphorylation; regulates Bcl-2 and Bax	(135)
GC	NSUN2	up	FOXC2	Promotes proliferation, migration, and invasion of GC cells	FOXC2-AS1 facilitates NSUN2 recruitment to FOXC2 mRNA, enhancing its m5C modification and interaction with YBX1	(96)
GC	NSUN2	up	NTN1	Promotes neural invasion in GC	DIAPH2-AS1 stabilizes NSUN2 and enhances the m5C modification of NTN1	(94)
CRC	NSUN2	up	SKIL	Promotes tumorigenesis and progression of CRC	Increases SKIL mRNA stability	(136)
HCC	NSUN5	up	ZEED3	Promotes proliferation of HCC cells	Activates Wnt/β-catenin signaling pathway	(137)
HCC	ALYREF	up	EGFR	Facilitates cell proliferation, invasion, and EMT in HCC	Induces m5C modification and increases the stabilization of EGFR mRNA and pSTAT3 activation.	(138)
HCC	NSUN2	up	SARS2	promotes the proliferation, colony formation, migration, and invasion of HCC cells	Mediates m5C of the SARS2 and activates the Wnt signaling pathway	(105)
AML	NSUN2	up	SRSF2	Increases the development of leukemia	Reduces NSUN2 expression lowers mRNA m5C levels, diminishes SRSF2 binding, and affects RNA splicing.	(43)
AML	YBX1	up	MYC and BCL2	Sustains the function of leukemia cells	Enhancing the stability of MYC and BCL2	(39)
AML	TET2		TSPAN13	Promotes leukemia development, leukemia stem cell migration/homing, and leukemia stem cell self-renewal	Increases the stability and expression of TSPAN13 transcripts	(110)
BLCA	NSUN2	up	HDGF	Promotes the proliferation and invasion of BLCA cells	Stabilizes HDGF mRNA	(139)
EC	NSUN2		SLC7A11	Promotes EC cell proliferation	Increased mRNA stability of SLC7A11	(113)
HNSC	NSUN2	up	TEAD1	Enhancing tumor cell proliferation and invasion of HNSC	Increased mRNA stability of TEAD1	(117)

Up, up-regulated in cancer.

**Table 2:** A summary of abbreviations and full names of different cancer types.

Abbreviation	Full Name	Abbreviation	Full Name
ACC	Adrenocortical Carcinoma	LUAD	Lung Adenocarcinoma
BLCA	Bladder Urothelial Carcinoma	LUSC	Lung Squamous Cell Carcinoma
BRCA	Breast Invasive Carcinoma	MESO	Malignant Mesothelioma
CESC	Cervical Squamous Cell Carcinoma	OV	Ovarian Serous Cystadenocarcinoma
CHOL	Cholangiocarcinoma	PAAD	Pancreatic Adenocarcinoma
COAD	Colon Adenocarcinoma	PCPG	Pheochromocytoma and Paraganglioma
ESCA	Esophageal Carcinoma	PRAD	Prostate Adenocarcinoma
GBM	Glioblastoma Multiforme	READ	Rectum Adenocarcinoma
HNSC	Head and Neck Squamous Cell Carcinoma	SARC	Sarcoma
KICH	Kidney Chromophobe	SKCM	Skin Cutaneous Melanoma
KIRC	Kidney Renal Clear Cell Carcinoma	STAD	Stomach Adenocarcinoma
KIRP	Kidney Renal Papillary Cell Carcinoma	TGCT	Testicular Germ Cell Tumors
LAML	Acute Myeloid Leukemia	THCA	Thyroid Carcinoma
LGG	Lower Grade Glioma	THYM	Thymoma
LIHC	Liver Hepatocellular Carcinoma	UCEC	Uterine Corpus Endometrial Carcinoma

### 6.1 Nervous system tumors

In gliomas, the expression of m5C methyltransferases varies with different clinical and pathological tumor characteristics. A risk prediction model constructed using five m5C methyltransferase genes can predict patient survival and clinical features in gliomas. Cox regression analysis has shown that the model’s risk score is an independent prognostic factor for gliomas (80). Additionally, in glioblastoma multiforme (GBM), most miRNAs exhibit m5C modification, with methylation of miRNA-181a-5p correlating with poor prognosis in GBM patients. Mechanistically, the m5C modification of miR-181a-5p, mediated by a complex containing DNMT3a and AGO4, inhibits the formation of miRNA-181a-5p/mRNA duplexes, resulting in the loss of its tumor-suppressive effects (81). In the U87 human glioma cell line, NSUN2 regulates tumor cell migration by modulating the autocrine chemokine (ATX)-lysophosphatidic acid (LPA) axis. NSUN2 methylates the cytosine at position 2756 in the 3’-UTR of ATX mRNA, enhancing ATX mRNA translation. The ATX-LPA pathway mediates cancer cell migration. Moreover, ALYREF interacts with methylated ATX mRNA, facilitating its export from the nucleus to the cytoplasm. NSUN2 knockout inhibits U87 cell migration, which can be restored by the addition of LPA (82). In *in vivo* glioma models, NSUN5 exhibits high methylation in CpG island promoter regions, leading to reduced transcript levels and epigenetic silencing. Silencing of NSUN5 induces a loss of methylation at the C3782 site of 28S rRNA. Under stress conditions, the unmethylated state results in a global depletion of protein synthesis while activating specific mRNA translation programs, leading to upregulation of NAD(P)H-quinone oxidoreductase 1 (NQO1) (83). NQO1 overexpression enhances sensitivity to NQO1-targeted drugs. Therefore, NSUN5 epigenetic silencing is considered a protective factor in gliomas and is associated with better prognosis.

### 6.2 Respiratory system tumors

#### 6.2.1 Lung cancer

In lung adenocarcinoma, two distinct m5C methylation modification patterns based on 11 m5C regulatory factors have been identified, each characterized by different tumor microenvironment immune cell infiltration profiles. A scoring system for m5C methylation modifications indicates that patients in the high-score group have better prognosis compared to those in the low-score group. A prognostic model constructed from 14 m5C-related lncRNAs shows that high-risk patients have poorer outcomes than low-risk patients, with high sensitivity and specificity. In lung squamous cell carcinoma (LUSC), the m5C regulatory factors NSUN3 and NSUN4 are highly expressed compared to normal lung tissue and are associated with poor prognosis (84). NSUN3 and NSUN4 expression is upregulated and correlates with adverse outcomes, and these factors are used to construct prognostic risk signatures. Additionally, NSUN3 and NSUN4 are related to the infiltration of six major immune cell types. In lung adenocarcinoma, *in vitro* experiments show that high expression of NOP2 or heterogeneous nuclear ribonucleoproteins (hnRNP) is more likely associated with poor differentiation. NSUN3 genomic deletions are common in non-smokers with lung adenocarcinoma, occurring at a rate of 15% (85). Research indicates that high expression of NSUN2 leads to resistance to gefitinib and promotes recurrence of lung cancer tumors. Knockdown of NSUN2 can overcome the intrinsic resistance of lung cancer cells to gefitinib. Mechanistic studies show that NSUN2 regulates the m5C modification of QSOX1, and YBX1 enhances QSOX1 translation in an m5C-dependent manner, thereby promoting resistance to EGFR-mutant lung cancer (2). THOC3 is highly expressed in lung squamous cell carcinoma (LUSC) and significantly promotes the growth, migration, and glycolysis of LUSC cells. Mechanistic studies have shown that THOC3 can form a complex with YBX1 to promote the transcription of PFKFB4. Additionally, THOC3 facilitates the export of PFKFB4 mRNA to the cytoplasm, while YBX1 maintains the stability of PFKFB4 mRNA (86). NSUN2 upregulates the m5C modification of NRF2, with YBX1 binding to the m5C-modified NRF2 to maintain its transcript stability, thereby promoting the proliferation, migration, and ferroptosis resistance of NSCLC cells (87).The expression level of NOP2 is abnormally elevated in lung cancer, and its increased expression enhances the migratory and invasive abilities of lung cancer cells, as well as the growth and metastasis of transplanted tumors. This effect is achieved by regulating the m5C modification level of EZH2 mRNA, which in turn stabilizes EZH2 mRNA through ALYREF mediation (88).Conversely, NSUN6 is downregulated in lung cancer, and overexpression of NSUN6 inhibits the proliferation, migration, and EMT of lung cancer cells. This is attributed to NSUN6 regulating the expression of NM23-H1 by m5C modification of NM23-H1 mRNA’s 3’-UTR (89). NC02159 is reported to be upregulated in the tumor tissues and serum of NSCLC patients, and knocking down LINC02159 significantly inhibits the proliferation, migration, and invasion of NSCLC cells, induces apoptosis and cell cycle arrest, and slows tumor growth *in vivo*. The primary mechanism involves interaction with Aly/REF export factor (ALYREF), thereby upregulating the stability of YAP1 mRNA in an m5C-dependent manner, activating the Hippo and β-catenin signaling pathways, and promoting NSCLC progression (37).

### 6.3 Digestive system tumors

#### 6.3.1 Esophageal cancer

`Esophageal cancer is highly aggressive with early metastatic potential and poor prognosis. Its two major histological subtypes are squamous cell carcinoma and adenocarcinoma. NSUN2-methylated lncRNA (NMR) is significantly upregulated in esophageal cancer tissues and is associated with reduced overall survival (90). Screening for genes with reduced m5C levels and sequencing analysis reveal that the m5C levels of migration and invasion-related genes PLOD3, COL4A5, LAMB1, and HSPG2 decrease following NMR overexpression. This reduction may be due to the competitive inhibition of mRNA m5C levels by the upregulated NSUN2 lncRNA. NSUN2 expression is positively regulated by E2F transcription factor 1 (E2F1), which enhances NSUN2 expression by binding to its promoter (91). This, in turn, increases the m5C levels of growth factor receptor-bound protein 2 (GRB2). The RNA-binding protein lin-28 homolog B (LIN28B) preferentially binds to m5C-modified GRB2 mRNA, stabilizing it. Subsequently, increased GRB2 levels activate phosphoinositide 3-kinase (PI3K)/protein kinase B (AKT) and extracellular regulated protein kinases (ERK)/mitogen-activated protein kinases (MAPK) signaling pathways, promoting the progression of esophageal squamous cell carcinoma (90). In esophageal cancer, RNA m5C methylation is primarily mediated by NSUN2 and participates in the disease process by affecting cancer-related genes and pathways. Recent studies have found that YBX1 is aberrantly overexpressed in esophageal squamous cell carcinoma (ESCC), with a significant correlation between high YBX1 levels and poor patient survival. YBX1 enhances the stability of spermine oxidase (SMOX) mRNA through an m5C-dependent mechanism mediated by NSUN2, promoting ESCC cell proliferation and metastasis (92).

#### 6.3.2 Gastric cancer

Studies indicate that NSUN2 expression is upregulated in gastric cancer compared to adjacent non-cancerous tissues. *In vitro* and *in vivo* experiments confirm that NSUN2 promotes gastric cancer cell proliferation and tumor development. RNA sequencing has identified p57KIP2 as a downstream target of NSUN2 regulation. Mechanistically, the methyltransferase activity of NSUN2 and m5C modification in the 3’-UTR region of p57Kip2 mRNA disrupts its stability, thereby facilitating gastric cancer progression (93). Research indicates that NSUN2 is upregulated in gastric cancer and is significantly associated with lower survival rates in patients. Functional studies reveal that NSUN2 methylates lncRNA-NR_033928, resulting in the upregulation of NR_033928. This lncRNA promotes the formation of the IGF2BP3/HUR complex, which subsequently maintains the stability of the downstream target gene GLS mRNA, leading to increased expression of glutaminase (GLS). This upregulation promotes gastric cancer (GC) cell proliferation and progression. NSUN2 has been reported to interact with lncRNAs to regulate the stability of target genes. In gastric cancer (GC) tissues, lncRNA-DIAPH2-AS1 is abnormally upregulated and is associated with poor prognosis in GC patients. Overexpression of DIAPH2-AS1 enhances the migration, invasion, and neural invasion potential of GC cells. Mechanistic studies have confirmed that DIAPH2-AS1 interacts with NSUN2, protecting NSUN2 from ubiquitin-proteasome degradation. This interaction further increases the stability of the downstream target gene NTN1 mRNA through m5C modification, ultimately inducing neural invasion in GC (94). Upregulation of m5C methyltransferases and binding proteins is observed in gastrointestinal cancers, and their high expression is significantly associated with poor patient survival (95). Bioinformatics analysis reveals that m5C regulatory proteins are closely related to the ErbB/PI3K-Akt signaling pathway, with GSK3B being a crucial target. FOXC2 antisense RNA 1 (FOXC2-AS1), a newly identified functional lncRNA, is highly expressed in gastric cancer tissues and cells, promoting cell proliferation, migration, and invasion, and correlates with poor prognosis. FOXC2-AS1 recruits NSUN2 to FOXC2 mRNA, increasing its m5C levels, and subsequently enhances FOXC2 mRNA stability through binding with m5C-binding protein YBX1 (96). Previous studies have shown that YBX1 is highly expressed in advanced gastric cancer tissues and is associated with shorter disease-free survival, though the exact mechanisms by which YBX1 promotes cancer progression through binding to RNA m5C methylation regions remain to be elucidated (96).

#### 6.3.3 Gallbladder cancer

In gallbladder carcinoma (GBC), NSUN2 expression is upregulated in both cells and tissues. Silencing of NSUN2 inhibits GBC cell proliferation and tumorigenesis, whereas overexpression of NSUN2 promotes gallbladder cancer cell growth. RPL6 contributes to carcinogenesis by regulating the translation of NSUN2 mRNA. In RPL6-silenced cells, NSUN2 protein levels are reduced, leading to the accumulation of NSUN2 mRNA (17).

#### 6.3.4 Colorectal cancer

Detecting tumor prognostic markers is crucial for identifying colorectal cancer patients with low survival rates and high mortality. Research has shown that in colorectal cancer patients and mouse models, the mRNA levels of NSUN5 and YBX1, as well as the total RNA m5C levels, are elevated (97). Co-culture experiments indicate that colorectal cancer cells promote the expression of NSUN5 and YBX1 in immune cells, leading to increased m5C levels in these cells. This suggests that m5C levels in peripheral blood immune cells may serve as potential biomarkers for distinguishing colorectal cancer patients. In colorectal cancer, NSUN2 suppresses miR-125b expression and enhances the expression of Grb-associated binding protein 2 (Gab2), thereby promoting cell migration (98). Additionally, NSUN5 is upregulated in colorectal cancer tissues and cells, and NSUN5 knockout mice exhibit significantly reduced cell proliferation and cell cycle arrest. NSUN5 may regulate colorectal cancer cell proliferation through the Retinoblastoma (Rb)-cyclin-dependent kinase (CDK) pathway (22). Bioinformatics analysis of differentially expressed genes between colon cancer and adjacent tissues has identified DNMT2, NSUN6, and ALKBH1 as prognostic genes for colorectal cancer, with all three involved in MAPK and P53 signaling pathways, suggesting their potential oncogenic roles (99). Elevated NSUN2 levels in colorectal cancer are associated with poor patient survival. Silencing NSUN2 inhibits tumorigenesis and progression in NSUN2 knockout mouse models, with mechanistic studies showing that NSUN2 induces m5C modification of SKIL and mediates SKIL mRNA stability through YBX1. Increased SKIL levels activate transcriptional coactivators with PDZ-binding motifs (TAZ), promoting colorectal cancer progression (100).

#### 6.3.5 Liver cancer

Comparative studies between hepatocellular carcinoma (HCC) and adjacent non-cancerous tissues have revealed that HCC exhibits significantly higher levels of m5C peaks in mRNA, with a broader distribution (101). In addition to coding RNAs, the frequency of m5C methylation and the number of methylated genes are also significantly higher in circRNA and lncRNA within HCC tissues compared to adjacent non-cancerous tissues (101). The presence of RNA m5C modifications promotes HCC progression, with elevated levels of m5C regulators NSUN4 and ALYREF correlating negatively with poor prognosis in HCC patients (102). Recent studies have shown that NSUN2 deficiency suppresses proliferation and migration in HepG2 liver cancer cells. Transcriptomic sequencing and bisulfite sequencing (Bis-Seq) have demonstrated a significant reduction in m5C methylation and gene expression of lncRNA H19 following NSUN2 loss. Mechanistically, lncRNA H19 is a specific target of the NSUN2 RNA methyltransferase, with m5C modification affecting H19 half-life and stability. The m5C-modified H19 can promote tumorigenesis through specific binding to the tumor protein G3BP1 (18). Elevated expression of NSUN5 is associated with reduced relapse-free and overall survival rates and predicts poor prognosis in hepatocellular carcinoma. NSUN5 mRNA and protein levels are upregulated in HCC tissues, and NSUN5 overexpression promotes HCC cell proliferation and migration. Bioinformatics analysis indicates a positive correlation between NSUN5 and ribosomal and translation-related genes in HCC (103). However, research on whether NSUN5 acts as a methyltransferase affecting RNA m5C levels in liver cancer remains unexplored. ALYREF is highly expressed in HCC cell lines, and its loss inhibits HCC cell proliferation. Gene knockout studies reveal that genes with differential methylation following ALYREF knockout bind to ALYREF protein, with their biological functions enriched in cell cycle and HCC pathways, suggesting that ALYREF may regulate HCC development through influencing target gene methylation levels (104). A recent significant study found that NSUN2 is significantly upregulated in hepatocellular carcinoma (HCC), and its high expression is closely associated with poor prognosis in HCC patients (105). Functional studies showed that knockdown of NSUN2 significantly inhibited the proliferation, colony formation, migration, and invasion of HCC cells. Further molecular mechanism analysis revealed that NSUN2 mediates m5C RNA modification of the SARS2 gene, which in turn activates the Wnt signaling pathway, promoting liver cancer progression (105). These findings provide new insights into the role of NSUN2 in HCC and highlight its potential as a therapeutic target.

#### 6.3.6 Pancreatic cancer

Pancreatic cancer is highly malignant, with an incidence rate nearly equal to its mortality rate and a poor prognosis. NSUN2 plays an enzymatic role in mediating m5C methylation enrichment in RNA within pancreatic cancer cells. Knockdown of NSUN2 in pancreatic cancer cells significantly downregulates m5C methylation levels (106, 107). In pancreatic cancer mouse models, NSUN2 expression is upregulated in cancer cells, and its knockdown slows the growth of pancreatic cancer spheroids. In contrast to normal pancreatic tissues, the protein level of NSUN6 is reduced in pancreatic cancer tissues (108). Overexpression of NSUN6 in pancreatic cancer cells inhibits cell proliferation, and low NSUN6 expression is associated with poor patient survival, indicating its potential as an independent prognostic factor for predicting recurrence and survival in pancreatic cancer (24). Contrary to findings in other cancers where m5C methyltransferases are often overexpressed, the reduced expression of NSUN6 in pancreatic cancer suggests it may act as a protective factor, though the role of NSUN6 in mediating RNA m5C modifications warrants further investigation.

### 6.4 Hematologic tumors

#### 6.4.1 Leukemia

In leukemia, NSUN1 specifically interacts with BRD4 and directly binds to the CTD-S2P of RNA polymerase II (RNA-pol II). In 5-azacytidine (5-AZA)-resistant leukemia cells, a unique NSUN1/BRD4/RNA-pol II CTD-S2P complex is formed, mediating the development of 5-AZA-resistant chromatin structures and contributing to 5-AZA resistance in leukemia. Conversely, NSUN3 and DNMT2 exhibit opposing effects on 5-AZA-sensitive leukemia cells. Mechanistically, the RNA-binding protein hnRNPK directly interacts with m5C methyltransferases NSUN3 and DNMT2, lineage-determining transcription factors GATA1 and SPI1/PU.1, and CDK9/PTEFb, forming a unique complex at nascent RNA sites, which ultimately results in a 5-AZA-sensitive chromatin structure (109). Comparative analysis of bone marrow samples from 5-AZA-resistant and -sensitive leukemia patients reveals significantly higher levels of m5C mRNA in the 5-AZA-resistant samples. The expression levels of hnRNPK, NSUN1, and BRD4 are associated with leukemia progression and contribute to 5-AZA resistance and tumor development (109). Research reports indicate that YBX1 maintains the survival of myeloid leukemia cells in an m6A-dependent manner, while having no effect on normal hematopoiesis. YBX1 interacts with m6A readers IGF2BPs through its conserved Cold Shock Domain (CSD) to indirectly bind m6A-modified mRNA, thereby enhancing the stability of apoptosis-related genes MYC and BCL2, which in turn sustains the function of leukemia cells (39). Recent research indicates that TET2 regulates the accumulation of 5-methylcytosine (m5C) modifications in TSPAN13 mRNA. These m5C modifications are specifically recognized by YBX1, which increases the stability and expression of TSPAN13 transcripts. This process promotes leukemia development, leukemia stem cell migration/homing, and leukemia stem cell self-renewal (110).

### 6.5 Genitourinary system tumors

#### 6.5.1 Bladder cancer

In bladder cancer, RNA bisulfite sequencing (Bis-Seq) has identified frequent m5C methylation in cancerous tissues compared to adjacent non-cancerous tissues. Most m5C methylation sites are located in mRNA, with high-methylation mRNA significantly enriched in carcinogenic pathways (111). Further research shows that NSUN2 and YBX1 are aberrantly elevated in bladder cancer tissues. The proto-oncogene heparin-binding growth factor (HDGF) mRNA is methylated by NSUN2, and YBX1 stabilizes HDGF mRNA by binding to m5C methylation sites and recruiting ELAVL1, thereby promoting tumor development (112). Results demonstrate that ALYREF regulates the splicing and stabilization of hypermethylated RABL6 and TK1 mRNAs in an m5C-dependent manner to enhance the proliferation and invasion of UCB cells (112).

#### 6.5.2 Endometrial cancer

Epigenetic enhancement mediated by H3K4me3 levels leads to significant upregulation of NSUN2 in endometrial cancer (EC). Upregulation of NSUN2 promotes EC cell proliferation, while NSUN2 knockdown significantly increases lipid peroxides and lipid ROS levels in EC cells, thereby enhancing sensitivity to ferroptosis. Mechanistically, NSUN2 enhances m5C modification of SLC7A11 mRNA and directly binds to m5C sites on SLC7A11 mRNA through YBX1, leading to increased mRNA stability and elevated SLC7A11 levels. Targeting the NSUN2/SLC7A11 axis can inhibit *in vivo* and *in vitro* tumor growth in EC cells by promoting lipid peroxidation and ferroptosis (113).

### 6.6 Breast cancer

In triple-negative breast cancer, overexpression of NSUN2 promotes cancer cell proliferation, migration, and invasion through Myc. NSUN6 regulates the mammalian sterile 20-like kinase 1 (MST1) target gene of Yes-associated protein 1 (YAP1), leading to osteoclast differentiation and breast cancer bone metastasis (114). In breast cancer cells and tissues, hypomethylation of NSUN2 DNA results in overexpression of NSUN2 mRNA and protein. Upregulation of NSUN2 promotes proliferation, migration, and invasion of breast cancer cells, while NSUN2 knockout inhibits these processes. In triple-negative breast cancer (TNBC), upregulated NSUN2 acts as an oncogenic factor, while downregulated NSUN6 functions as a tumor suppressor. NSUN2 and NSUN6 influence tumorigenesis and the tumor immune microenvironment (TIM) in breast cancer. NSUN2/YBX1 synergistically upregulate HGH1 mRNA stability and promote breast cancer progression (115).

### 6.7 Neck squamous cell carcinoma

In head and neck squamous cell carcinoma (HNSCC), NSUN2 expression is significantly upregulated, which may be associated with mitochondrial function and cell cycle checkpoint-related genes. Additionally, DNA cytosine-5-methyltransferase 1 (DNMT1) is downregulated in HNSCC, potentially related to peptide cross-linking and humoral immunity. There is a negative correlation between NSUN2 expression and T cell activation scores (116). Furthermore, in hypopharyngeal squamous cell carcinoma (HPSCC), increased levels of NSUN3 enhance tumor proliferation and invasion. In HPSCC, both NSUN2 mRNA and protein levels are elevated. NSUN2 modifies the 3’-UTR of TEA domain transcription factor 1 (TEAD1) mRNA through m5C, promoting TEAD1 expression and thereby enhancing tumor cell proliferation and invasion (117). TEAD1 coordinates and integrates multiple signaling pathways, and its downregulation affects the expression of various oncogenes involved in tumor cell progression, metastasis, and resistance to chemotherapy.

## 7 The role of m5C in the tumor immune microenvironment and cancer immunotherapy

### 7.1 The role of m5C in the tumor immune microenvironment

The tumor immune microenvironment (TME) is closely associated with tumor progression and responses to immunotherapy. Recent studies have revealed that m5C modification regulates immune cell infiltration within tumors. TET2 and ten-eleven translocation 3 (TET3) play crucial roles in Treg cell immune homeostasis (118). Additionally, several m5C regulatory proteins within TME can serve as prognostic and diagnostic biomarkers for cancer. In lung adenocarcinoma, patients with high m5C scores have better prognoses, and different m5C modification patterns indicate varying immune infiltration profiles (119). Research indicates that m5C risk scores positively correlate with neutrophils, resting CD4+ memory T cells, and M2 macrophages in lung squamous cell carcinoma, while negatively correlating with follicular helper T cells, CD8+ T cells, and activated NK cells (120). The impact of m5C on TME is also increasingly recognized in other cancers. Multiple studies have demonstrated that m5C modifications are involved in regulating TME in HNSCC (121). Knockdown of NSUN3 has been shown to regulate M1/M2 polarization of macrophages in HNSCC, increasing M1 macrophage infiltration and inhibiting HNSCC growth both *in vitro* and *in vivo*. Moreover, 28S rRNA methyltransferase NSUN5 downregulates β-catenin by promoting CTNNB1 mRNA degradation, thereby enhancing the phagocytic activity of tumor-associated macrophages (TAMs). Interestingly, NSUN5 directly interacts with CTNNB1 chromatin-associated RNA (caRNA) and deposits m5C (21). Findings reveal that the content of resting NK cells, M2 macrophages, and neutrophils in the low-risk group is significantly lower than in the high-risk group. Additionally, m6A/m5C/m1A-related lncRNAs are associated with the immune microenvironment and tumor mutation burden in HNSCC, providing potential prognostic markers for immunotherapy in this cancer (116).

### 7.2 The role of m5C in cancer immunotherapy

Significant advancements have been made in the basic and clinical research of m5C-related cancer immunotherapy. On one hand, study successfully induced apoptosis and immunogenic cell death in cancer cells by combining m5C inhibitors with immune checkpoint inhibitors (122). These effects are associated with endogenous antitumor immune responses and the conversion of cold immune tumors to hot ones. On the other hand, mechanisms of m5C methylation modification have been employed to enhance the efficacy of mRNA-based immunotherapy. Research has shown that m5C methylation reduces RNA antigenicity and suppresses immune responses. Following methylation modification, the immunogenicity of RNA diminishes or disappears, thus avoiding activation of the innate immune system. This represents a novel breakthrough in RNA-based immunotherapy. Accordingly, m5C/m1C combinatorial modifications have been utilized to enhance the ability of exogenous mRNA to evade Toll-like receptor activation and downstream innate immune signaling, thereby improving protein expression from mRNA. Biotechnological teams have designed materials to deliver m5C-modified mRNA for reprogramming tumor-associated macrophages or anticancer T cells, thereby inducing antitumor immunity and promoting tumor regression.

**Figure 2:**
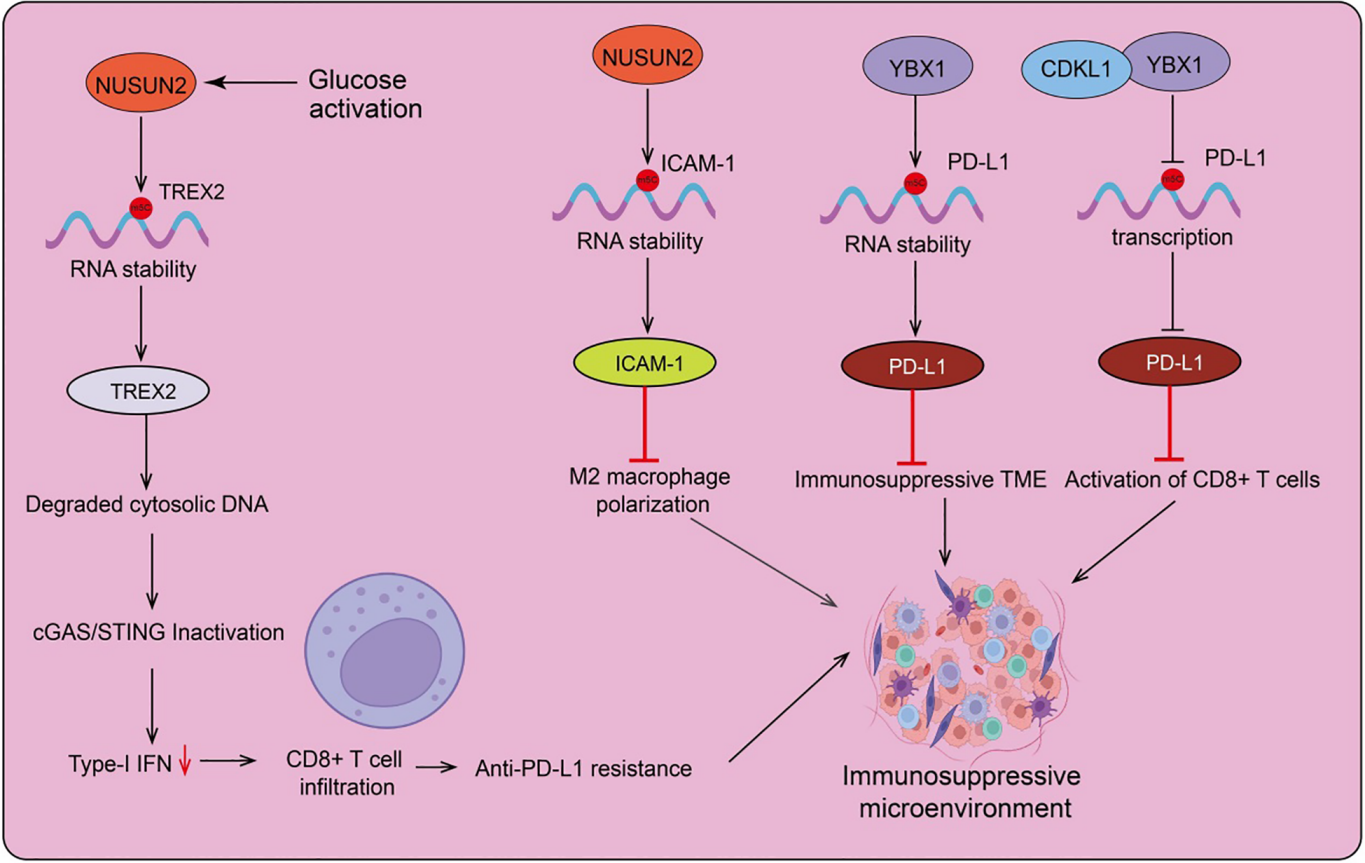
The role and mechanisms of RNA m5C modification in the regulation of the tumor microenvironment. NUSUN2 mediates m5C modifications of various downstream target genes, recruits the YBX1 reader protein, and subsequently regulates the RNA stability of these target genes, upregulating their expression. This plays a key role in tumor immune microenvironment remodeling, including M2 macrophage polarization, the formation of an immunosuppressive microenvironment, and CD8+ cell activation.

In head and neck squamous cell carcinoma (HNSCC), NSUN2 is negatively correlated with M2 macrophage polarization and T cell activation. Consequently, NSUN2 is considered a potential target for immune checkpoint blockade in HNSCC (123). Furthermore, NSUN2 negatively regulates immune cell infiltration in the nasopharyngeal carcinoma (NPC) tumor microenvironment, suggesting that NSUN2 may be inversely related to sensitivity to immunotherapy and chemotherapy. NSUN2 could be a significant oncogene involved in NPC progression. Recent research indicates that glucose, acting as a signaling molecule, directly binds to NSUN2 at its amino acid residues 1-28, causing NSUN2 oligomerization and activation, and sustaining m5C RNA methylation independent of glucose metabolism (16). Glucose, as a standalone signaling molecule, can directly bind and activate NSUN2, leading to tumorigenesis and immune therapy resistance by inhibiting the cGAS/STING pathway (12). The glucose/NSUN2/TREX2 axis drives tumorigenesis and resistance to PD-L1 immune therapy in immune-competent syngeneic tumor mouse models by suppressing the cGAS/STING pathway, apoptosis, and CD8+ T cell infiltration. Notably, gene targeting of the glucose/NSUN2/TREX2 axis reduces tumorigenesis and overcomes resistance to PD-L1 immune therapy by promoting the cGAS/STING pathway, apoptosis, and CD8+ T cell infiltration (**Figure 2**). This research provides foundational evidence that targeting the glucose/NSUN2/TREX2 axis is a promising strategy for overcoming resistance to PD-1/PD-L1 immune therapies in cold tumors, offering a basis for converting prostate cancer and other cold tumors into hot tumors that respond to PD-1/PD-L1 immune therapy (16).

Luo et al. discovered that NSUN2 enhances the expression of ICAM-1 by upregulating m5C methylation in ICAM-1 mRNA, which improves the adhesion between leukocytes and endothelial cells, and inhibits M2 macrophage polarization and suppresses tumor metastasis (124) (**Figure 2**). Additionally, the absence of donor NSUN2 impedes the development of atherosclerosis in a rat model of allogeneic aortic transplantation, suggesting that the NSUN2-ICAM-1 regulatory axis is involved in endothelial cell inflammation. Beyond ICAM-1 mRNA, NSUN2 can also catalyze the methylation of other mRNAs and non-coding RNAs. Therefore, further research is needed to elucidate the mechanisms by which NSUN2 regulates vascular inflammation and the development of atherosclerosis (124) (**Figure 2**). Studies indicate that chemotherapy induces an immunosuppressive microenvironment within tumors and promotes immune evasion through YBX1-mediated upregulation of PD-L1 (programmed death-ligand 1) (125). Knocking out YBX1 reverses chemotherapy resistance by blocking PD-L1 expression and activating T cells in the tumor microenvironment. The upregulation of functional cytotoxic CD8+ T cells, and the downregulation of myeloid-derived suppressor cells and regulatory T cells, are associated with overcoming tumor immunosuppressive environments and immune evasion (126). Additionally, YBX1 knockout can reverse hepatocellular carcinoma (HCC) resistance by blocking PD-L1 expression and activating T cells in the tumor microenvironment (127). CDKL1 is highly expressed in lung cancer and promotes the growth and proliferation of lung cancer cells, while also enhancing their radiosensitivity. Further studies have discovered that CDKL1 interacts with YBX1, thereby inhibiting YBX1-mediated transcription of the PD-L1 gene and suppressing PD-L1 expression. This ultimately leads to the activation of CD8+ T cells and the inhibition of lung cancer immune evasion. Increased expression of CDKL1, combined with radiotherapy and anti-PD-L1 antibody therapy, can significantly improve the therapeutic outcomes for lung cancer (128) (**Figure 2**).

The development of m5C regulatory protein and lncRNA-related risk models also provides new insights for cancer treatment and efficacy prediction, enabling more accurate and personalized immunotherapy regimens. The m5C risk score serves as an independent prognostic factor for colon cancer patients, with lower scores indicating greater sensitivity to immunotherapy and higher scores indicating greater sensitivity to chemotherapy (129). This score can predict colon cancer prognosis, immunotherapy response, and drug sensitivity. These immunotherapy prediction methods are also applicable to other cancers. In triple-negative breast cancer (TNBC), changes in the expression of m5C RNA methylation regulators, with upregulation of NSUN2 and downregulation of NSUN6, can significantly predict clinical prognosis risk in TNBC patients. Therefore, it may serve as a new prognostic marker for TNBC and provide insights into RNA epigenetic modifications in TNBC (130). Related studies have also confirmed that NSUN3 and NSUN4 can predict the prognosis of lung squamous cell carcinoma and regulate the immune microenvironment. In lung adenocarcinoma patients, different m5C patterns correlate with variations in TME immune cell infiltration, with high m5C scores associated with better prognosis. Additionally, m5C-regulated lncRNAs can predict overall survival in lung adenocarcinoma patients and impact the tumor immune microenvironment (131). In pancreatic cancer patients, three m5C-related lncRNAs show prognostic value. The TIDE (Tumor Immune Dysfunction and Exclusion) algorithm indicates that patients with high m5C-lncRNA scores respond better to immunotherapy. In another study on pancreatic ductal adenocarcinoma (PDAC) (107), researchers evaluated the relationship between m5C-related lncRNAs and PDAC-infiltrating immune cells. Naïve B cells, CD8+ T cells, Treg cells, and resting NK cells were more highly expressed in the low-risk group, while M0 and M2 macrophage phenotypes were more highly expressed in the high-risk group, suggesting that m5C-related lncRNAs may regulate pancreatic cancer progression by promoting M2 macrophage polarization or infiltration in PDAC (107).

## 8 Summary and future directions

Current research has provided preliminary insights into the distribution characteristics of m5C methylation across various RNAs and the biological functions of m5C modifications. Future research efforts should primarily focus on elucidating the roles of specific m5C methylation sites, discovering new recognition proteins, and understanding the precise roles of m5C modifications in diseases such as cancer. Significant progress has already been made in elucidating the protein crystal structures of certain m5C methylation enzymes and recognition proteins, as well as their RNA-binding domains. The development of inhibitors targeting m5C methylation-related enzymes has become a focal point of research. Furthermore, several studies have suggested that m5C-related modification enzymes could serve as diagnostic biomarkers for cancer. However, the use of specific m5C modification sites as cancer biomarkers still requires further investigation. Overall, the regulatory role of m5C methylation in tumorigenesis is gradually being uncovered, offering new perspectives for cancer diagnosis and personalized treatment.

Notably, RNA m5C methylation modifications have shown significant potential in cancer immunotherapy. Research indicates that modulating m5C methylation levels can enhance antitumor immune responses and improve the efficacy of immune checkpoint inhibitors. For instance, inhibiting the function of m5C-related proteins such as NSUN2 or ALYREF can restore T cell antitumor activity and enhance the effects of immunotherapy (16, 36). Therefore, in-depth studies on the mechanisms of RNA m5C methylation modifications and their applications in cancer immunotherapy are of substantial clinical significance. In summary, the functions and mechanisms of RNA m5C methylation modifications in neurodevelopment, autoimmune diseases, and cancer progression hold significant research value and application potential. Future research aiming to further elucidate the mechanisms of m5C modifications and their regulatory pathways is expected to reveal additional biological processes and advance their application in disease diagnosis and treatment.

To improve and develop techniques for detecting m5C (5-methylcytosine) modifications in RNA, various innovative sequencing technologies have been explored, such as Nanopore-seq and single-molecule real-time (SMART) sequencing (132, 133). These technologies aim to overcome limitations of traditional methods, offering advantages in terms of sensitivity, real-time analysis, and the ability to detect modifications at single-molecule resolution. Nanopore sequencing is an emerging technology that can directly sequence nucleic acids by passing them through a protein nanopore, which detects changes in the ionic current as the nucleotides translocate through the pore. However, challenges still exist in the high error rates associated with Nanopore sequencing, particularly for short sequences, and distinguishing between m5C and other modifications or sequence-context effects can be difficult. Efforts to improve base-calling algorithms and modify the sequencing technology to improve its accuracy are ongoing. SMART sequencing, pioneered by Pacific Biosciences (PacBio), is another promising technique for detecting RNA modifications like m5C (133). SMART sequencing relies on real-time observation of the DNA polymerase activity during the sequencing process. In summary, Nanopore-seq and SMART sequencing represent exciting advances in the detection of RNA modifications like m5C, offering significant advantages over traditional sequencing technologies. As these techniques continue to evolve, they hold the potential to provide a more comprehensive, accurate, and real-time understanding of RNA modification dynamics, furthering our understanding of RNA biology and its implications in health and disease.

The application of Nanopore-seq and single-molecule real-time (SMART) sequencing in detecting m5C modifications holds significant promise for advancing our understanding of RNA epigenetics. These cutting-edge technologies offer unprecedented sensitivity and resolution for identifying m5C modifications at a single-base level, enabling researchers to explore m5C’s dynamic role in gene regulation and disease processes. Future research should focus on optimizing these sequencing techniques for high-throughput, cost-effective detection of m5C across different cell types and tissues, particularly in the context of cancer and other diseases. Additionally, integrating Nanopore-seq and SMART with other omics technologies, such as transcriptomics and proteomics, could provide a comprehensive view of how m5C modifications interact with other epigenetic marks to regulate cellular functions. This integrated approach could pave the way for discovering novel biomarkers and therapeutic targets, ultimately improving our ability to diagnose and treat diseases driven by aberrant m5C regulation.

The targeting of m5C modification in the tumor immune microenvironment presents a promising avenue for future cancer research. As recent studies have shown, m5C modifications play a critical role in regulating gene expression and immune responses within tumors, potentially influencing tumor progression and immune evasion. Understanding the mechanisms by which m5C modification regulates immune cells, such as T cells, macrophages, and dendritic cells, could open up new strategies for enhancing anti-tumor immunity. Future research should focus on identifying specific m5C-modifying enzymes, exploring their interactions with immune checkpoints, and investigating how m5C modification can be harnessed to modulate the immune microenvironment. Additionally, combining m5C-based therapies with immune checkpoint inhibitors may offer synergistic effects, improving therapeutic outcomes. This emerging field holds great potential for developing novel cancer immunotherapies, offering hope for more effective and personalized treatments.
